# Injury Patterns in Collegiate Club Quidditch

**DOI:** 10.1186/s40798-021-00336-4

**Published:** 2021-06-26

**Authors:** Christopher M. Fox, Jonathan G. Wu, Lucia Chen, Dena L. Florczyk

**Affiliations:** 1 University of Missouri-Kansas City School of Medicine and Truman Medical Centers, Kansas City, MO USA; 2 Harbor-UCLA Medical Center Department of Internal Medicine, 1000 W. Carson St, Torrance, CA 90502 USA; 3 UCLA Division of General Internal Medicine and Health Services Research, Los Angeles, CA USA; 4 UCLA Arthur Ashe Student Health and Wellness Center, UCLA Department of Sports Medicine, Los Angeles, CA USA

**Keywords:** Quidditch, Epidemiology, Club sports, Injury, Concussion, Surveillance, College, Club, Sports

## Abstract

**Background:**

The purpose of this study is to assess injury patterns in collegiate club quidditch athletes at a single university over three seasons. Injury data were gathered from athletic trainers that provided sideline medical coverage during competitions, the on-site athletic training center where athletes had daily access for evaluation and treatment for acute and chronic injuries, and a sports medicine physician at the on-campus student health center. Athlete exposures were estimated using available previous rosters, practice, and game schedules for the 2014–2017 quidditch seasons. Injuries were evaluated regarding the sex of the athlete, mechanism, body part injuries, and injury type. This is a retrospective descriptive epidemiology study.

**Results:**

The overall injury incidence rate (IR) for collegiate club quidditch injuries was 4.55 per 1000 athlete exposures (AEs). Male athletes had an IR = 5.22 (95% CI 3.77, 7.23). Females had an IR = 3.77 (95% CI 2.49, 5.72). The most common mechanism of injury in males was collision with another athlete (36%; IR = 1.88; 95% CI 1.09, 3.24). The most common injuries were lower extremity injuries (foot, ankle, lower leg, knee, thigh, hip/groin) at 57%. The most common injury type in males was sprains at 39% (IR = 2.03; 95% CI 1.20, 3.42). The overall incidence rate for all quidditch athletes for concussions was 1.18 per 1000 AEs.

**Conclusions:**

Quidditch is an increasingly popular mixed-gender collegiate club sport. This study helps identify areas for improvement in education, injury prevention, and care of athletes at the local and national levels. Concussion rates in quidditch are comparable to other contact sports and should encourage discussion to make rule changes to improve the safety of the sport.

## Key Points

- The most common injuries in club quidditch were lower extremity injuries.
- The most common injury in club quidditch among males seen by medical professionals was sprains.
- Concussion rates in club quidditch are comparable to those in other full-contact sports.

## Background

Quidditch is a relatively new sport that is becoming increasingly popular in the USA as well as around the world. It is a sport based on a fictional game from the Harry Potter book series which was first published in 1997 by JK Rowling. The first quidditch game ever played in the USA was in 2005 at Middlebury College in Vermont [1]. In 2010, US Quidditch (USQ) became an official nonprofit organization and by 2012 there were over 100 teams registered with USQ [1]. Quidditch now involves many national teams including a biannual World Cup, which was last held in Florence, Italy, in 2018 with 29 teams competing [2].

The rules of the game have been adapted from the fictional game in the Harry Potter book series. A unique aspect to quidditch involves its rules regarding gender and contact documented under the term Title 9 ¾ [3]. According to USQ, each team is allowed to have a maximum of four players who identify as the same gender on the field at once [3]. Quidditch is also considered a mixed-gender contact sport [4]. There are seven athletes on the pitch or field per team who must keep a broomstick between their legs at all times. The pitch is an oval-shaped field that is 66 × 36 yards across. There are four positions per team and each one is designated with a different color headband. These positions include one “keeper” who guards their team’s three hoops. There are three “chasers” who are trying to throw a “quaffle” through any one of the other team’s three hoops for 10 points. The quaffle is the name of the volleyball used in the game. There are also 2 “beaters” who throw “bludgers” at the chasers to disrupt them from scoring goals. The bludgers are spherical balls made of flexible rubber such as dodgeballs. The last player is the “seeker” who chases and attempts to catch the “snitch” for 30 points. The “snitch” is a tennis ball held in a sock that is dangling from the back of the shorts of the snitch runner. Catching the snitch also ends the game [4]. There are fouls in the game which lead to different consequences depending on the severity and involve time in the penalty box.

Quidditch is becoming increasingly popular on US college campuses. According to the USQ website, there are currently 210 college teams registered in the USA as of 2021 [5]. US collegiate quidditch is competitive and popular among students; however, there is limited medical coverage designated for these events. Quidditch has been compared to rugby due to its full-contact nature and lack of body padding [6]. Given the unique aspects of quidditch being a mixed-gender full-contact sport and requiring the use of athletes to hold a broomstick at all times, we would expect a unique distribution of injuries. There is limited data on quidditch injury incidence rates and types.

The current level of research around quidditch is minimal. In a recent search on PubMed, there were only 3 articles related to quidditch—one retrospective study and one prospective study looking at incidence rates of injuries in competitive quidditch [7]. One study on quidditch looked at injuries in the UK assessed by a retrospective self-administered survey given to athletes registered with the national governing body asking about injuries over the previous 12 months [7]. The incidence rates were reported as injury per 1000 h of play and found an overall incidence rate of 4.06 per 1000 h [7]. Although the study had participants respond to a questionnaire reporting injuries that were evaluated by first responders, nurses, paramedics, and doctors, the study is limited by recall bias. Since data collection was based on a self-reported survey, injuries were not necessarily reported by a medical professional. To improve reporting accuracy, our study instead had all injuries and concussions diagnosed by a medical professional. However, if an athlete did not present to the club sport athletic trainer or was seen by an outside physician, it may have been missed in our review. A prospective study by Brezinski on club collegiate quidditch in one season found an incidence rate of 16.2 (95% CI 6.1, 26.2). Although this study was similar to our study by having healthcare professionals diagnose and record injuries by healthcare professionals, it only consisted of one season and resulted in a smaller sample and less athlete exposures (AE = 619) [8].

The purpose of our study is to assess the injury patterns in collegiate club quidditch athletes at a single university over three seasons and add to the current literature on its injury patterns.

## Methods

This is a retrospective descriptive epidemiology study taken place at a single, large, public university located in Los Angeles, California. This study was evaluated by our university Institutional Review Board (IRB) and determined to not need IRB approval since our data was de-identified and the data fell under the purview of the Family Educational Rights and Privacy Act (FERPA) since it relates to students.

The injury data originated from male and female athletes participating in practices and competitions while representing the home university’s lone collegiate club quidditch team. Injuries that occurred to athletes from visiting club quidditch teams on the university campus during competitions were not included in the data.

There is an athletic training center staffed by certified athletic trainers (ATCs) on this university campus. The training center is open in the afternoon and evening during the hours when most club practices are held. There were ATCs present to provide sideline medical coverage at quidditch competitions and tournaments. By having ATCs available during competitions, athletes could be evaluated and treated at the time of injury. If additional care was needed, the athlete was referred to a sports medicine doctor at the campus student health center for additional imaging and treatment.

Injury information from practices and competitions was recorded by the selected certified athletic trainer and inputted into the club sport athletic training database, Sports Injury Monitoring System (SIMS) (FlanTech Inc, Iowa City, IA). The injury information was gathered from SIMS and the university student health electronic medical records. As determined by our IRB committee, consent was not required to access injury data since this was a retrospective epidemiological study based on chart review and the patient’s data was de-identified. Data was clean, ordered, and statistically analyzed in Microsoft Excel for Microsoft 365 MSO (version 16.0.13426.20352; Microsoft Corp, Redmond, WA).

An athlete exposure (AE) is defined as a collegiate club athlete participating in one club organized practice or competition where the participant may have been “exposed to the possibility of injury, regardless of time associated with participation” [9]. AEs were estimated using rosters, practice, and game schedules for the 2014–2017 quidditch seasons.

We define injury as (1) “tissue damage or other derangement of normal physical function due to participation in sports, resulting from rapid or repetitive transfer of kinetic energy,” (2) a result of participation in organized club practice or competition, (3) required medical attention by a certified athletic trainer or medical provider, and (4) resulted in the restriction of the athlete from 1 or more calendar days beyond the day of the injury [10–12]. Despite data collection being conducted prior to the publication of the IOC’s 2020 consensus statement, our definition for injury happened to be consistent with that detailed in the consensus statement [13]. Each injury was recorded only once. Athletes who returned for follow-up for the same condition were not counted as a separate case. The percentage of injuries is expressed as the proportion of all reported injuries.

Overuse is defined as an injury that occurs “with gradual onset over time and result from a mechanism of repetitive stress and cumulative trauma” [14]. Non-contact is defined as an injury without disruption or impedance of the athlete’s movement [13]. Contact is defined as forces that originate from contact with another athlete, ground, or ball [9]. Injury data were not reported by mode of onset, mechanism, body regions, body areas, type, severity, and training subcategories listed in the IOC’s 2020 consensus statement because this study’s procedure and data collection was started and completed before the release of the consensus statement [13].

Injury data were evaluated by sex, injury type, injury mechanism, and body part. Body parts were listed as head/face, neck, upper arm/shoulder/clavicle, lower arm/elbow, hand/wrist, trunk, hip/groin, thigh, knee, lower leg, ankle, and foot. Injury incidence rates (IRs) were calculated by reported injuries per 1000 total AEs. Incidence rate ratios (IRRs) compared men versus women. The α level was set at .05. We calculated all 95% confidence intervals (CIs) using standard formulae for Poisson rates [15]. Ranges not containing 1.00 were considered statistically significant.

## Results

There were 135 athletes on the quidditch roster over three seasons. There were slightly more males (71) compared to females (64) participants. There was a total of 58 injuries that required evaluation for an IR of 4.55 per 1000 AEs, 36 injuries among males and 22 injuries among females (Table 1). Accounting for all practices and games over three seasons for male participants, there were 6900 AEs which produced IR = 5.22 (95% CI 3.77, 7.23) (Table 1). This is slightly higher than women participants who had 5835 AEs which produced IR = 3.77 (95% CI 2.49, 5.72) during the same time period (Table 1). Although IR for injuries occurring in men was 38% greater than women (IRR = 1.38; 95% CI 0.81, 1.24), it was not significant at the α = .05 level (Table 1).

**Table 1:** Injury incidence, athlete exposures (AEs), injury incidence rates, and injury rate ratios by sex in college club quidditch

	Injury incidence	AEs	IR per 1000 AEs^a^ 95% CI	Injury rate ratio^b^ 95% CI
Total	58	12735	4.55 (3.52, 5.89)	N/A
Sex				
Men	36	6900	5.22 (3.77, 7.23)	1.38 (0.81, 1.24)
Women	22	5835	3.77 (2.49, 5.72)	N/A

*Abbreviation*: *N/A* not applicable

^a^Incidence rates per 1000 athlete exposures for men’s and women’s team

^b^Incidence rate ratios for men’s/women’s team

The mechanism of injury, injury location, and type are provided in Tables 2, 3, and 4, respectively. Although this was not significant at the α = .05 level, the most common mechanism of injury in all athletes was collision with another athlete at 29% (IR = 1.33; 95% CI 0.83, 2.15) (Table 2). When this was evaluated for differences in sex, the most common mechanism of injury in males remained to be collision with another athlete (36%; IR = 1.88; 95% CI 1.09, 3.24). The most common injury mechanism in females was non-contact (32%; IR = 1.20; 0.57, 2.52), but was also not significant at the α = .05 level (Table 2).

**Table 2:** Injury mechanisms^a^

Mechanism of injury	AE 12735			Men (AE 6900)			Women (AE 5835)			
	Total no.	% of total	IR per 1000 AEs 95% CI	No.	% of men	IR per 1000 AEs 95% CI	No.	% of women injury	IR per 1000 AEs 95% CI	Incidence rate ratio 95% CI
Collision with balls	2	3%	0.16 (0.04, 0.63)^b^	2	6%	0.29 (0.07, 1.16)	0	0%	0.00	N/A
Collision with ground	11	19%	0.86 (0.48, 1.56)	7	19%	1.01 (0.48, 2.13)	4	18%	0.69 (0.26, 1.83)	1.48 (0.43, 5.06)
Collision with other athletes	17	29%	1.33 (0.83, 2.15)	13	36%	1.88 (1.09, 3.24) ^b^	4	18%	0.69 (0.26, 1.83)	2.75 (0.90, 8.43)
Non-contact	15	26%	1.18 (0.71, 1.95)	8	22%	1.16 (0.58, 2.32)	7	32%	1.20 (0.57, 2.52)	0.97 (0.35, 2.67)
Overuse	8	14%	0.63 (0.31, 1.26)	4	11%	0.58 (0.22, 1.54)	4	18%	0.69 (0.26, 1.83)	0.85 (0.21, 3.38)
Unknown	5	9%	0.39 (0.16, 0.94)^b^	2	6%	0.29 (0.07, 1.16)	3	14%	0.51 (0.17, 1.59)	0.56 (0.09, 3.37)
Total	58	100%		36	100%		22	100%		

*Abbreviation*: *N/A* not applicable

^a^Incidence rates per 1000 athlete exposures for men’s and women’s team, incidence rate ratios for men’s/women’s team

^b^Incidence rate ratio significant at the α = .05 level

**Table 3:** Injury locations^a^

			AE 12735	Men (AE 6900)			Women (AE 5835)			
Body part	Total	% of total	IR per 1000 AEs 95% CI	No.	% of men	IR per 1000 AEs 95% CI	No.	% of women	IR per 1000 AEs 95% CI	Incidence rate ratio M/W
Ankle	9	16%	0.71 (0.37, 1.36)	3	8%	0.43 (0.14, 1.35)	6	27%	1.03 (0.46, 2.29)	0.42 (0.11, 1.69)
Lower arm/elbow	1	2%	0.08 (0.01, 0.56)	1	3%	0.14 (0.02, 1.03)	0	0%	0.00	N/A
Foot	5	9%	0.39 (0.16, 0.94)	2	6%	0.29 (0.07, 1.16)	3	14%	0.51 (0.17, 1.59)	0.56 (0.09, 3.37)
Hand/wrist	7	12%	0.55 (0.26, 1.15)	7	19%	1.01 (0.48, 2.13)	0	0%	0.00	N/A
Head/face	15	26%	1.18 (0.71, 1.95)	8	22%	1.16 (0.58, 2.32)	7	32%	1.20 (0.57, 2.52)	0.97 (0.35, 2.67)
Hip/groin	1	2%	0.08 (0.01, 0.56)	0	0%	0.00	1	5%	0.17 (0.02, 1.22)	N/A
Knee	15	26%	1.18 (0.71, 1.95)	11	31%	1.59 (0.88, 2.88)	4	18%	0.69 (0.26, 1.83)	2.33 (0.74, 7.30)
Lower leg	1	2%	0.08 (0.01, 0.56)	1	3%	0.14 (0.02, 1.03)	0	0%	0.00	N/A
Neck	0	0%	0.00	0	0%	0.00	0	0%	0.00	N/A
Upper arm/shoulder/clavicle	3	5%	0.24 (0.08, 0.73)	3	8%	0.43 (0.14, 1.35)	0	0%	0.00	N/A
Thigh	1	2%	0.08 (0.01, 0.56)	0	0%	0.00	1	5%	0.17 (0.02, 1.22)	N/A
Trunk	0	0%	0.00	0	0%	0.00	0	0%	0.00	N/A
Total	58	100%		36	100%		22	100%		

*Abbreviation*: *N/A* not applicable

^a^Incidence rates per 1000 athlete exposures for men’s and women’s team, incidence rate ratios for men’s/women’s team

**Table 4:** Injury types^a^

Type			AE 12734	Men (AE 6900)			Women (AE 5834)			
	Total	Total %	IR per 1000 AEs	No.	% of men	IR per 1000 AEs	No.	% of women	IR per 1000 AEs 95% CI	Incidence rate ratio M/W
ACL tear	2	3%	0.16 (0.04, 0.63)^b^	1	3%	0.14 (0.03, 1.33)	1	5%	0.17 (0.02, 1.22)	0.85 (0.05, 13.52)
Bone stress injury	0	0%	0.00	0	0%	0.00	0	0%	0.00	N/A
Bursitis	0	0%	0.00	0	0%	0.00	0	0%	0.00	N/A
Concussion	15	26%	1.18 (0.71, 1.95)	8	22%	1.16 (0.58, 2.32)	7	32%	1.20 (0.57, 2.52)	0.97 (0.35, 2.66)
Contusion	5	9%	0.39 (0.16, 0.94)^b^	3	8%	0.43 (0.14, 1.35)	2	9%	0.34 (0.09, 1.37)	1.27 (0.21, 7.59)
Dislocation	2	3%	0.16 (0.04, 0.63)^b^	2	6%	0.29 (0.07, 1.16)	0	0%	0.00	N/A
Fracture	2	3%	0.16 (0.04, 0.63)^b^	2	6%	0.29 (0.07, 1.16)	0	0%	0.00	N/A
Laceration	0	0%	0.00	0	0%	0.00	0	0%	0.00	N/A
Impingement	1	2%	0.08 (0.01, 0.56)^b^	1	3%	0.14 (0.02, 1.03)	0	0%	0.00	N/A
Meniscus tear	2	3%	0.16 (0.04, 0.63)^b^	1	3%	0.14 (0.02, 1.03)	1	5%	0.17 (0.02, 1.22)	0.85 (0.05, 13.52)
PTFS	3	5%	0.24 (0.08, 0.73)^b^	1	3%	0.14 (0.02, 1.03)	2	9%	0.34 (0.09, 1.37)	0.42 (0.04, 4.66)
Sprain	19	33%	1.49 (0.95, 2.34)	14	39%	2.03 (1.20, 3.42) ^b^	5	23%	0.86 (0.36, 2.06)	2.37 (0.85, 6.57)
Strain	1	2%	0.08 (0.01, 0.56)^b^	0	0%	0.00	1	5%	0.17 (0.02, 1.22)	N/A
Tendonitis	6	10%	0.47 (0.21, 1.05)	3	8%	0.43 (0.14, 1.35)	3	14%	0.51 (0.17, 1.59)	0.85 (0.17, 4.19)
Total	58	100%		36	100%		22	100%		

*Abbreviation*: *N/A* not applicable

^a^Incidence rates per 1000 athlete exposures for men’s and women’s team, incidence rate ratios for men’s/women’s team

In all athletes, the most common injuries were lower extremity injuries (foot, ankle, lower leg, knee, thigh, hip/groin) at 57%. The most common injuries in males and females were lower extremity injuries at 48% and 69%, respectively.

For males, the most common injury location was the knee (31%; IR = 1.59) followed by the head/face (22%; IR = 1.16), although both were not statistically significant at the α = .05 level (Table 3). For females, the most common injured body was also the head/face (32%; IR = 1.20) followed by the ankle (27%; IR = 1.03), although both were also not statistically significant at the α = .05 level (Table 3).

Injuries were also evaluated by the type of injury. The most common injury type in males was sprains at 39% (IR = 2.03; 95% CI 1.20, 3.42) (Table 4). The most common injury type in females was concussions at 39% (IR = 1.20); however, it was not statistically significant at the α = .05 (Table 4). The overall incidence rate for all athletes for concussions was 1.18 per 1000 AEs. Based on our data, there were no IRRs that were statistically significant at the α = .05 level.

## Discussion

When comparing the injury incidence rates found with collegiate club quidditch to other NCAA championship collegiate sports, the incidence rates were less than other sports. According to a study by Kerr et al., the overall incidence rate for injuries in NCAA men’s sports was 6.5 per 1000 AEs and 5.2 per 1000 AEs in female sports [16]. Our study demonstrated men and women quidditch athletes had lower injury incidence rates compared to the NCAA data. This is possible due to lack of institutional support as a club sport and results in less competitions and practices and therefore less opportunities for injuries. However, this lack of institutional support can also increase the risk of injury. Since collegiate quidditch is a club sport and self-funded by the club athletes, quidditch athletes lack nutritional, medical, and coaching support available to NCAA championship teams.

Injuries to the lower extremity (including foot, ankle, leg, knee, thigh, hip/groin) accounted for 57% of all injuries. The proportion of lower extremity injuries is more than reported in a previous study by Pennington et al. that found 33% of injuries located in the lower extremity [7].

Our injury incidence rate is much lower compared to a prospective study by Brezinski who found an injury incidence rate of 16.2 (95% CI 6.1, 26.2) per 1000 AEs [8]. Brezinski found 10 injuries in 619 athlete exposures. The large difference between our incidence rate and Brezinski highlights the need for more studies to be conducted to accurately determine the true injury rate in club quidditch. Unfortunately, our study which reported injures per 1000 athlete exposures is unable to make an accurate comparison with Pennington et al.’s study which reported their injury incidence rate as injuries per 1000 h of quidditch played.

Concussions are “defined as a traumatically induced transient disturbance of brain function” and continue to be a common sport-related injury [17]. It is associated with severe short-term and long-term health consequences if not properly medically managed. Concussions tend to occur at a high rate in contact sports. NCAA men’s lacrosse had a reported incidence rate of concussions at 0.3 per 1000 AEs [18]. The game concussion rate for collegiate football was 3.74 per 1000 AEs in a study from 2015 by Dompier et al. [19]. In our study, the incidence rate for concussions was 1.18 per 1000 AEs for all athletes. In the literature, there is a high rate of under-reporting of concussions by athletes across a range of contact sports and this would likely apply to quidditch [17, 20]. Therefore, this study’s incidence rate of concussions is likely under-reported because of decreased athlete disclosure and the authors did not have medical providers provide sideline coverage at practices which can be another setting where concussions can occur. The proportion of concussions reported by Pennington et al.’s is also likely under-reported since data was collected from a self-reported survey and subject to inaccuracy [7]. Of note, at the university where this study occurred, as part of university protocol to help clinicians determine return to play from concussions, all quidditch players are required to complete ImPACT (https://impacttest.com/) testing prior to participation.

In our study, 26% of all injuries were reported as concussions (*n* = 15). This is a similar percentage of injuries to Pennington et al.’s study that reported 21.5% of injuries were concussions [7]. Our study also found a higher percentage of concussions occurring in females compared to males which is similar to Pennington et al.’s study who reported a statistically significant difference in the rate of concussions observed between males and females with females experiencing more [7]. This is consistent to findings in the literature that found concussion rates are higher among females than males which can be due to various reasons including differences in disclosure, neck musculature, cerebral blood flow, hormonal regulation, among others [17, 21]. The findings in Pennington et al.’s study and our study about the difference between males and females could be related to the full-contact and mixed-gender nature of the sport; however, further prospective studies are needed for better assessment as there is limited research on injury rates related to mixed-gender sports. One limitation of our study was that we did not categorize the injury mechanism of the concussions if they were due to contact with another person, contact with the surface, or contact with equipment. With the unique aspect of quidditch having athletes holding a PVC pipe between their legs, throwing balls at opponents, and permitting of contact, there are multiple causes of concussions in this sport that need further investigation.

In our current study, all injuries and concussions were diagnosed by a medical professional; however, if an athlete did not present to the club sport athletic trainer or was seen by an outside physician, it may have been missed in our review. Some athletes may not present to a provider after an injury, and this may lead to an underestimation of the incidence rates reported above. We were also unable to calculate time lost due to injury with the data we had available, and AEs were estimated based on the team roster, practice, and event schedules. There remains a good opportunity for a prospective study in the future to better assess these incidence rates as well as time lost due to injury.

## Limitations

There were several limitations in this research article. One limitation was that the authors did not utilize all the reporting guidelines recommended by the IOC’s 2020 consensus statement because the study’s data collection protocol and analysis took place prior. Unfortunately, this will make this data difficult to compare to future studies that follow the 2020 IOC statements. Another limitation was the diagnoses were not specific enough to use an ICD disease classification system. If ICD diagnosis was implemented, it would allow for comparisons with injury data between college quidditch and data from the NCAA Injury Surveillance System [9]. Additionally, the authors did not demonstrate the severity of injury by not reporting the amount of time missed from practice and games due to injury. This study will not be able to compare the time loss in college quidditch with other sports in the NCAA Injury Surveillance System and other studies. This study did not report if the injuries occurred in practice or competition and which sport position experienced injuries and therefore cannot determine which factors result in a higher risk in injuries.

## Conclusions

This study provides information on injury rates and patterns for an increasingly popular and mixed-gender sport. It also helps identify areas for improvement in education, injury prevention, and the care of athletes. We are seeing concussion rates similar to other contact sports and our current studies may actually be under-reporting this incidence rate. As this sport is played on more college campuses, it may require policies to ensure proper oversight of these athletes. We hope our epidemiologic information will help athletic trainers and college campuses plan for events and provide adequate medical coverage. Hopefully, this study’s findings and others can encourage the sport’s national governing body to institute rule changes that eliminate behaviors that increase concussion risk, provide more information and education to recognize the symptoms of concussions, and improve the safety of this sport.

## Data Availability

The datasets used and/or analyzed during the current study are available from the corresponding author on reasonable request.
